# Induced neuroprotection by remote ischemic perconditioning as a new paradigm in ischemic stroke at the acute phase, a systematic review

**DOI:** 10.1186/s12883-020-01836-8

**Published:** 2020-07-02

**Authors:** Francisco Purroy, Cristina García, Gerard Mauri, Cristina Pereira, Coral Torres, Daniel Vazquez-Justes, Mikel Vicente-Pascual, Ana Vena, Gloria Arque

**Affiliations:** 1 Stroke Unit, Department of Neurology, Universitat de Lleida, Hospital Universitari Arnau de Vilanova, Avda Rovira Roure 80, 25198 Lleida, Spain; 2 Clinical Neurosciences Group, Institut de Recerca Biomèdica de Lleida (IRBLleida). UdL, Lleida, Spain

**Keywords:** Ischemic stroke, Neuroprotection, Remote ischemic perconditioning, Randomized clinical trials, Systematic review

## Abstract

**Background:**

Remote ischemic conditioning during cerebral ischemia (remote ischemic perconditioning, RIPerC) refers to the application of several cycles of brief ischemia and reperfusion (I/R) commonly to a limb, and it represents a new paradigm in neuroprotection with multiple mechanisms of action in ischemic stroke (IS) patients during acute phase. Some clinical trials just finished, and a few others are still ongoing; gather the current knowledge and pull it down to influence the present and future studies was the goal of this paper.

**Methods:**

A systematic review of published research papers and/or registered clinical trials since 2000 was performed.

**Results:**

Nineteen studies were identified and only four studies were completed. All of them have demonstrated that RIPerC is safe, feasible and well tolerated in IS patients. However, a high heterogeneity of clinical trial characteristics was observed: five (26.3%) randomized clinical trials (RCTs) included only thrombolytic-treated patients, three (15.8%) RCTs only thrombectomy-treated patients, and five (26.3%) RCTs required radiological confirmation of IS. Temporal inclusion criteria vary from 4 h to 48 h. Most of the clinical trials used 4 cycles of RIPerC in the upper non-affected limb. Interestingly, only three (16.7%) RCTs applied RIPerC during the transportation in the ambulance. Neuroimaging outputs were the main endpoints when endovascular therapy was applied; functional outcome is also the main endpoint in large-medium size studies.

**Conclusions:**

This review summarizes the completed and ongoing clinical trials on RIPerC in IS patients, where RIPerC has been used alone or in combination with recanalization therapies. Ongoing clinical trials will provide new information on the best RIPerC intervention strategy and potentially improve the functional outcome of IS patients; definition of new RIPerC strategies would ideally aim at enhancing tissue preservation, promoting neurological recovery, and stratify patients to improve treatment feasibility.

## Background

Stroke is one of the leading causes of death and disability worldwide [1], with 10.3 million of new strokes and 113 million of disability-adjusted life years per year [2]. Stroke victims face an uncertain future and a life severely affected by disability. The most common type of stroke is the ischemic stroke (IS), accounting for 87% of all strokes. It is characterized by the occlusion within an arterial vessel supplying blood to an area of the brain, resulting in a corresponding loss of neurological function. It mainly occurs in elderly patients of both sexes with often multiple comorbidities (diabetes mellitus, hypertension, hyperlipidemia, obesity) [3]. Currently, the only treatments available in the acute phase that have demonstrated safety and effectiveness are intravenous fibrinolytic treatment [4, 5] and mechanical thrombectomy [6]. Unfortunately, even today many patients cannot benefit from these treatments due to contraindications, time of evolution of the symptoms or restricted access to mechanical therapies that are currently only offered in highly sophisticated hospitals. Thus, there is a need for better and wider therapies to boost patient adherence.

The effectiveness of neuroprotective therapies has a great potential to not only increase the benefits of available reperfusion therapies but also to provide an advisable medical procedure for patients who are not eligible for current treatments. However, translation of most neuroprotective trials from the bench to the emergency room has failed so far, they did not demonstrate efficacy on IS patients, even with promising results in a few preclinical studies [7]. One of the main explanations for this failure is that the majority of neuroprotective drugs studied only act on a level of the complex cascade of phenomena that occur in ischemia/reperfusion [7–9]. The feasibility of neuroprotection in IS is still an unresolved inquiry. To date, all trials of neuroprotectant compounds have failed to provide basis and build better trials.

**Fig. 1:**
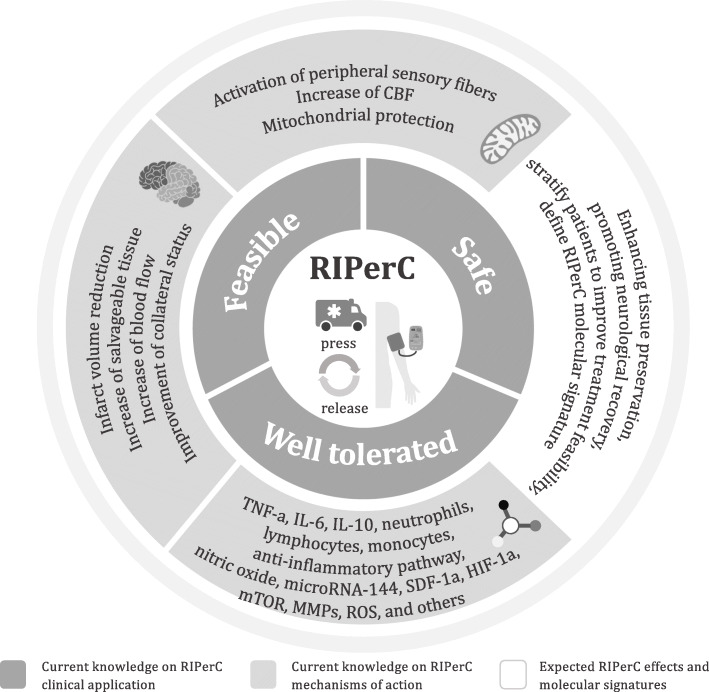
Schematic diagram of the potential and expected neuroprotective effects of remote ischemic perconditioning (RIPerC) on ischemic stroke at the acute phase. RIPerC refers to the application of several cycles of press and release by an automatic device in a prehospital setting (ambulance) to an upper non-affected limb. Its clinical application is safe, feasible and well tolerated. The underlying RIPerC mechanisms include mitochondrial protection, activation of inflammasome, neurovascular protection and specific anti-inflammatory pathway regulation. Ongoing clinical trials will provide new information on the best RIPerC intervention strategy and reveal underlying neuroprotective mechanisms. Abbreviations: CBF, cerebral blood flow; TNF-a, tumor necrosis factor alpha; IL-6, interleukin 6; IL-10, interleukin 10; SDF-1a, stromal cell-derived factor-1; HIF-1a, hypoxia-inducible factor 1-alpha; mTOR, mammalian target of rapamycin; MMPs, matrix metallopeptidases; ROS, reactive oxygen species

Remote ischemic perconditioning (RIPerC) represents a new paradigm in neuroprotective therapies [10, 11] and it has the potential ability to protect the ischemic brain from injury until reperfusion and, later to protect the brain from reperfusion injury. RIPerC consists of short and controlled cycles of ischemia-reperfusion applied to one limb during the establishment of cerebral ischemia [11]. Until now, the underlying mechanisms of remote ischemic conditioning (RIC) include neurovascular protection, induced anti-inflammatory action and neuronal protection against excitotoxicity; paired together with mitochondrial protection, circulating inflammasome activation and/or transcriptional regulation of neuroprotective pathway [12] (Fig. 1). However, there is limited data about the clinical translation of RIPerC in IS patients.

Endogenous cerebral neuroprotection of RIPerC on IS patients is a new paradigm that aims to enhance the brain resilience to ischemia and it has the potential to improve the clinical outcome of affected individuals. For that, a systematic review of the published articles and clinical trials on RIPerC applied to IS patients was performed to evaluate and summarize the findings.

### Search strategy and selection criteria

A systematic review of prospective cohort studies was conducted (prehospital-based and hospital-based cohorts) on acute IS patients under RIPerC and placebo-arms. Studies published from January 2000 to March 2020 were included. PRISMA recommendations were followed [13].

Identification, screening and eligibility for included studies was performed by two reviewers (F.P., G.A.). Bias analysis was unable to be performed because of the ongoing clinical trials. The search was conducted using the electronic databases: Pubmed and ClinicalTrials.gov. Search limits were English language, human and 2000-current. The search terms were: ‘remote ischemic conditioning’ AND ‘acute ischemic stroke’ OR ‘remote ischemic perconditioning’ AND ‘acute ischemic stroke’ OR ‘remote ischemic postconditioning’ AND ‘acute ischemic stroke’. Prospective human cohort studies that applied RIPerC in IS patients were included. Studies accepting inclusion beyond 48 h from the onset of symptoms were excluded. The last database search was conducted on September 2019. Following screening of abstracts, full-text copies of potentially eligible papers were retrieved and assessed for eligibility.

## Results

**Fig. 2:**
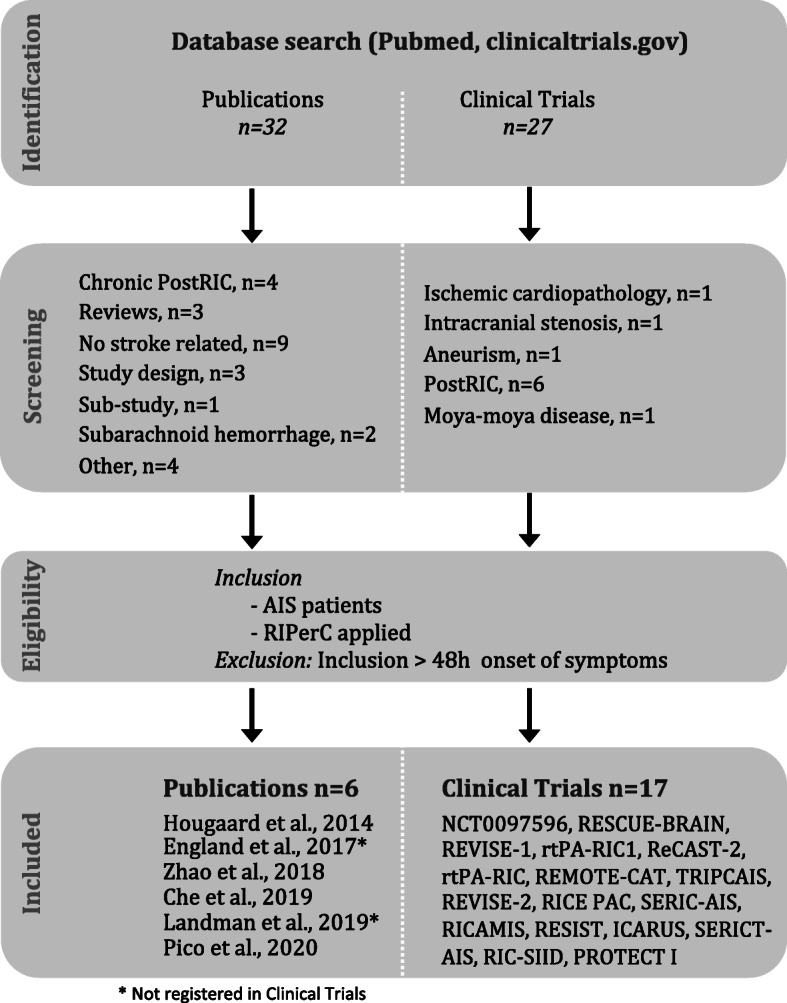
PRISMA diagram details the search and selection process of RIPerC systematic review of literature

Electronic database search yielded 32 publications and 27 clinical trials of which 19 studies were finally included in the systematic literature review (Fig. 2). Among the 31 publications identified on Pubmed search, nine articles were not related to stroke (29%), four articles applied chronic PostRIC (13%), three articles were reviews of literature, three articles described design of the studies or protocols [14–16], two articles were on subarachnoid hemorrhage patients, one article was a sub-study and four articles were not eligible. After applying the inclusion criteria (acute ischemic stroke-AIS patients and application of remote ischemic perconditioning-RIPerC) and the studies that accept inclusion beyond 48 h from the onset of symptoms were exclude; a total of 6 articles were included and analyzed in the systematic review [16–21], note that 4 out of the 6 papers were previously registered as clinical trials [18–21]. Twenty-seven randomized clinical trials (RCTs) were identified on clinicaltrials.gov. Of these RCTs, 6 (22.2%) applied PostRIC and 4 (14.8%%) were not considered after inclusion/exclusion criteria were applied. Seventeen RCTs were further considered in the present systematic review (NCT0097596 [21], RESCUE-BRAIN [15, 22], REVISE-1 [18], rtPA-RIC1 [19], ReCAST-2, rtPA-RIC, REMOTE-CAT, TRIPCAIS, REVISE-2, RICE PAC, SERIC-AIS, RICAMIS, RESIST [14], ICARUS, SERICT-AIS, RIC-SIID, PROTECT I). Table 1 provides a summary of study design characteristics of the 19 RTCs on RIPerC application on IS patients.

**Table 1:** Summary of characteristics of 19 clinical trials and research papers included in this systematic review on remote ischemic per-conditioning application on acute ischemic stroke patients

NTC Number Acronym/Ref	Country	Registered year	Estimated enrollment	Age	Criteria	Time before inclusion	Prehospital intervention	Application # cycles Pressure	Application of RIC	Intervention	Primary outcome measures	Recruitment status
NCT0097596 [21]	Denmark	2009	120	> 18	Ischemic stroke and rtPA therapy candidate, cerebral infarct showed on MRI	4.5 h	Yes	Manual rIPerC, 4 × 5′ Up to 25 mmHg above systolic BP	Non-affected upper extremity	One time Control group: not RIC simulation	Difference in infarct growth (PWI-DWI) after 24 h (Salvage index)	Completed and published
NCT02189928 RESCUE-BRAIN [15, 20]	France	2014	200	> 18	Carotid ischemic stroke, confirmed by MRI, NIHSS ≥5 and ≤ 25	< 6 h	No	Device, 4 × 5′ 110 mmHg	Non-affected lower extremity	One time after initial MRI Control group: RIC simulation (no pressure)	Infarct volume by MRI at 24 h mRS at 90 days	Completed and published
RECAST [17]	UK	2015^a^	26	> 18	Ischemic stroke with motor deficits on arm and/or leg	< 24 h	No	Manual RIC, 4 × 5′ Up to 20 mmHg above systolic BP	Non-affected upper extremity	One time Control group: manual RIC simulation (30 mmHg)	Tolerability and feasibility	Complete and published
NCT03210051 REVISE-1 [18]	China	2017	20	18–80	Ischemic Stroke and endovascular recanalization	< 6 h	No	Device, 5 × 5′ 200 mmHg	Both upper extremities	One time No control group	Frequency of adverse events at 90 days	Completed
NCT03231384 rtPA-RIC1 [19]	China	2017	30	> 18	Confirmed Ischemic Stroke and rtPA therapy on going	< 4.5 h	No	Doctormate device, 5 × 5′ 200 mmHg	Both upper extremities	One time 2 h after rtPA therapy; twice daily for 6 days. Control group: not RIC simulation	Feasibility of RIC within 7 days	Completed
NCT02779712 ReCAST-2	UK	2016	120	> 18	Ischemic stroke	6 h	No	Manual, 4 × 5′ Up to 20 mmHg above systolic BP	Non-affected upper extremity	Group1: one time. Group2: two times (repetition after 60 min). Group3: twice daily for 4 days. Control group: manual RIC simulation (30 mmHg)	Trial feasibility at 90 days	Completed
NCT02886390 rtPA-RIC	China	2016	60	18–80	Clinical sign and symptoms of acute ischemic stroke and rtPA therapy candidate, NIHSS score ≥ 4 and ≤ 15	4.5 h	No	Doctormate device, 5 × 5′ 200 mmHg	Both upper extremities	One time within 2 h after rtPA therapy Control group: not RIC simulation	Infarct volume Infarct volume by MRI at 72 h	Recruiting
NCT03375762 REMOTE-CAT	Spain	2017	572	> 18	Clinical signs and symptoms of acute ischemic stroke, RACE > 0, RACE motor > 0, known-onset stroke	< 8 h	Yes	Device, 5 × 5′, 200 mmHg	Non-affected upper extremity	One time in the ambulance Control group: RIC simulation (no pressure)	Infarct volume by MRI at 72 h mRS at 90 days	Recruiting
NCT03218293 TRIPCAIS	China	2017	120	all	Confirmed ischemic stroke by neuroimaging, accordance with GTAIS and accomplish rtPA therapy	< 4.5 h	No	RIPC Device, 5 × 5’	Non-affected upper extremity	One time after rtPA therapy Control group: not RIC simulation	VEGF and bFGF levels at 14 and 90 days	Recruiting
NCT03045055 REVISE-2	China	2017	180	18–80	Confirmed Ischemic Stroke, NIHSS ≥6, Endovascular recanalization	< 6 h	No	Device, 4 × 5′ 200 mmHg	Upper extremity	One time Control group: RIC simulation (60 mmHg)	Infarct volume at 3–7 days post-stroke	Not recruiting yet
NCT03152799 RICE PAC	UK	2017	60	> 18	Ischemic Stroke, proximal anterior occlusion, endovascular recanalization	< 6 h	No	Manual	Non-affected upper/lower extremity	One time Control group: RIC simulation	Infarct volume by MRI at 90 days	Not recruiting yet
REPOST [16]	Netherlands	2017^b^	200	> 18	Ischemic stroke	< 12 h	No	Manual 4 × 5′ Up to 20 mmHg above systolic BP	Upper extremity	Twice daily × 4 days Control group: twice daily × 7 days (50 mmHg)	Infarct volume by MRI at 4 days	Recruiting
NCT03669653 SERIC-AIS	China	2018	912	18–80	Confirmed Ischemic Stroke, NIHSS score > 5 and ≤ 25	< 12 h	No	Device, 4 × 5′ 200 mmHg	Both upper extremities	Twice daily × 7 days Control group: twice daily × 7 days (60 mmHg)	mRS at 90 days	Recruiting
NCT03740971 RICAMIS	China	2018	1800	> 18	Confirmed Ischemic Stroke by neuroimaging, NIHSS score ≥ 6 and ≤ 16	48 h	No	–	–	Twice one day Control group: not RIC simulation	Neurological score at 90 days	Recruiting
NCT03481777 RESIST [14]	Denmark	2018	1500	> 18	Clinical signs and symptoms of stroke, PreSS ≥1	< 4 h	Yes	Device, 5 × 5’, 200 mmHg	Non-affected upper extremity	Two times, one at the ambulance and one 6 h after in the hospital. Some patients get twice daily for 7 days. Control group: RIC simulation (20 mmHg)	mRS at 90 days	Recruiting
NCT03481205 ICARUS	US	2018	10	18–85	Ischemic stroke, air transportation to a Stroke unit for endovascular recanalization, NIHSS ≥6	–	No	Doctormate Device, 3–5 × 5′ 200 mmHg	Both upper extremities	One time in route (airplane) to Stroke center No control group	Feasibility of delivering RLIC by air medical crews	Not recruiting yet
NCT04027621 SERICT-AIS	China	2019	50	18–80	Confirmed Ischemic Stroke and rtPA therapy, NIHSS score > 5 and ≤ 25	–	No	Device, 4 × 5′ 200 mmHg	Non-affected upper extremity	Twice within 6–24 h from rtPA therapy, Control group: twice within 6–24 h from rtPA therapy (60 mmHg)	Frequency of adverse events at 7 days or earlier	Not recruiting yet
NCT04069546 RIC-SIID	China	2019	30	> 18	Confirmed Ischemic Stroke, NIHSS ≤15	< 48 h	No	Device, 5 × 5′ 180 mmHg	Upper extremity	One time < 48 h from stroke symptom onset Control group: not RIC simulation	Plasma levels of mHLA-DR at 2 and 7 days, pneumonia incidence within 7 days	Not recruiting yet
NCT03915782 PROTECT I	France	2019	126	> 18	Ischemic Stroke, full occlusion of the MCA (occlusion of M1 and/or proximal M2), confirmed by MRA and DWI Endovascular recanalization	< 6 h	No	Device, 4 × 5′ 200 mmHg	Upper extremity	One time after first MRI Control group: RIC simulation (30 mmHg)	Infarct volume by MRI after 24 h from endovascular recanalization	Not recruiting yet

*Abbreviations*: *bFGF* Basic fibroblast growth factor, *DWI* Diffusion weighted imaging, *GTAIS* Guideline of thrombolysis in Acute Ischemic Stroke, *MCA* Middle cerebral artery, *MRI* Magnetic resonance imaging, *mRS* Modified Rankin Scale (mRS) Score, *NIHSS* National institute of Health Stroke Scale, *PreSS* Prehospital Stroke score, *PWI-DWI* Perfusion-weighted imaging-diffusion-weighted imaging, *RACE* Rapid arterial occlusion evaluation scale, *RIC* Remote ischaemic conditioning, *RLIC* Remote limb ischemic conditioning, *rt-PA* Recombinant tissue plasminogen activator, *VEGF* Vascular endothelial growth factor

^a^Registered in ISRCTN

^b^Registered in Netherlands Trial Register

The first research paper was published by Hougard et al. in 2014 [21]. Of 443 randomized patients, 247 received manual remote ischemic conditioning (mRIC) during transportation in the ambulance to the hospital. After adjustment for baseline multimodal magnetic resonance imaging (MRI) findings, voxel-wise logistical analysis showed better radiological evolution of mRIC treated patients than non-treated patients. However, there were no significant differences in clinical neurological outcome between mRIC and control groups. The paper of Che et al. [19], included only 30 patients treated with recombinant tissue plasminogen activator (rt-PA). Zhao et al. [18] demonstrated that RIC is safe in 20 patients who underwent mechanical thrombectomy. Moreover, England et al. [17] confirmed the applicability and feasibility of RIC on 13 IS patients within 24 h after the onset of symptoms. Furthermore, RIC was associated with changes of plasma biomarkers related to ischemic tolerance (IT) phenomena, such as HSP27 and phosphorylated HSP27, whose expression was significantly different when both arms (control vs. experimental) of the trial were compared (*n* = 13) [17]. These four publications included a limited and small number of recruited subjects [17–19, 21]. In contrast with previous studies, the multicenter RESCUE-BRAIN trial [20] was not only focused on IS patients who received or were candidate for revascularization therapies. It included 188 patients with confirmed carotid IS who underwent magnetic resonance imaging within 6 h after the onset of symptoms, and 171 (91%) patients received a recanalization therapy. In RESCUE_BRAIN trial, RIPerC was applied using an electronic device on the unaffected lower extremity (4 cycles of 5-min inflations and 5-min deflations). Brain infarction volume growth, which was the main outcome, was not significantly different between the intervention and control groups. In addition, no significant differences at 90-days mRS and mortality were observed between the two groups.

Up to now, there are 19 RCTs identified (where?) and 17 (89.5%) of them were registered in clinicaltrials.gov. Among them, 14 (73.4%) have been registered in the last 3 years, 9 (47.4%) have been developed in China, 9 (47.4%) in Europe and one (5.3%) in United States. Relating to the estimated number of enrolled patients on selected RTCs, special attention must be paid on RICAMIS (*n* = 1800), RESIST (*n* = 1500, 14), SERIC-AIS (*n* = 912) and REMOTE-CAT (*n* = 572).

There is a high variability in the inclusion and exclusion criteria among trials. Five RCTs require radiological confirmation of acute cerebral infarction despite of the subsequent treatment received (SERIC-AIS, RIC-SHD, RICAMIS, RECAST, RESCUE BRAIN). Finally, Danish RESIST RCTs, Spanish REMOTE-CAT and British RECAST-2 include patients that met stroke code criteria. Both REMOTE-CAT and RESIST consider the score of prehospital scales: RACE scale [23] and Prehospital Stroke Score (PreSS), respectively. Only 6 trials (31.6%) set up an upper age limit as an inclusion criterion. Like in previous RCTs of Hougard et al. [21] and Che et al. [19], three on-going RCTs (SERICT-AIS, rtPA-RIC, TRIPCAIS) are focused on the RIC’s role as an adjuvant treatment of thrombolytic therapy. In contrast, REVISE-2, PROTECT I and REVISE-1 [18] included patients who underwent thrombectomy.

Heterogeneity is also evidenced by the number of RIC cycles applied: 7 (36.8%) RCTs use 5 cycles, one (5.3%) RCT uses between 3 and 5 cycles, and the rest of the trials use 4 cycles. Thirteen (68.4%) RCTs perform a single application of RIC. Conversely, SERIC-AIS and RESIST [14] have planned up to two applications throughout 7 days, like in the finished study of Che et al. [19]; only REPOST has planned to applied during 4 days [16]. The application of RIC is located in the non-paretic lower limb only in one RCT [15], on both upper extremities in five (26.3%) RCTs, and on upper or lower non-paretic extremities in one (5.3%) RCT. In most cases, the application is restricted to the unaffected upper limb. The application of the RIC is manual in 5 (26.3%) RCTs: two completed RCT [17, 21], REPOST [16], RECAST 2 and RICE PAC. A simulated control group is only included in little over half of the considered RCTs.

Certain variability of timing of RIC application is observed within all selected studies. Concretely, in the RESIST trial, temporal inclusion criterion is set at < 4 h while in RIC-SIID and RICAMIS is extended to 48 h. RCTs focused on patients treated with intravenous fibrinolysis set the maximum time for the evolution of symptoms to 4.5 h. Instead, among RCTs assessing the effect of RIC on thrombectomy, the time is set up at 6 h. The Spanish REMOTE-CAT trial includes patients with less than 8 h of evolution of symptoms.

Only three RCTs, REMOTE-CAT, RESIST and the previous published by Hougard et al. [21], initiate the application of RIC in a prehospital setting, usually in the ambulance transportation of the patient to the hospital or stroke care center. Despite the low sample size (*n* = 15), ICARUS trial aims to reveal the feasibility of RIC application on thrombectomy candidates who are transported to comprehensive stroke centers by aircraft.

C, outcome measurements, was there any information on the size of the final infarct volume, perfusion, recurrent stroke?

**Fig. 3:**
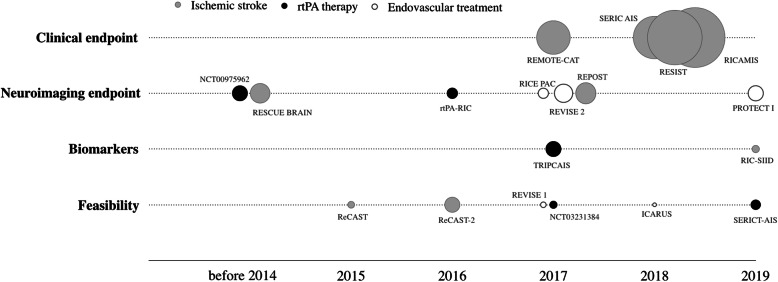
Forest plot of included clinical trials and research papers summarized by ischemic stroke (grey dots), rt-PA therapy (black dots) and endovascular treatment (white dots). Dots height is proportional to estimated enrollment. The analysis included data from 18 studies on four variables: clinical endpoints, neuroimaging endpoint, biomarker discovery and feasibility

The high heterogeneity within RCTs is also observed on the main endpoints (Fig. 3) and outcome measurements. The RCTs yielding the highest number of enrolled patients are still on-going (REMOTE-CAT, SERIC AIS, RESIT and RICAMIS) and all have considered the clinical endpoint as the main endpoint. In medium size studies and endovascular therapy related studies, the main endpoints are infarct volume and/or neuroimaging outputs. On the first research published paper on the application of RIC on IS patients, the main endpoint considered was the neuroimaging outcome [21]. Ischemic tolerance-related biomarkers are included in TRIPCAIS and RIC-SIID trials. However, other RCTs would also study biomarkers to detect differential expression changes. Small-size recruited patients studies demonstrate whether RIC application is feasible in AIS patients and AIS patients treated with rt-PA and/or endovascular therapy [17–19] (Fig. 3).

## Discussion

The current systematic review of RIPerC in IS patients has revealed a noticeable number of trials registered in clinicaltrials.gov, especially in the last 3 years. Globally, a broad heterogeneity is observed among RCTs regarding the number of recruited patients, inclusion criteria, number of RIPerC applied cycles, location of the application, and the main endpoints. Despite the high heterogeneity of current studies, they would all contribute to improve RIPerC effects and mechanisms of action. The first published evidence of RIPerC in IS patients was limited to patients that underwent intravenous alteplase therapy [21]. Moreover, according to new advances in stroke, five new studies have been focused on patients treated with endovascular therapy. However, preclinical data have demonstrated that RIC during acute ischemia is effective when applied both alone and in combination with revascularization therapies [24]. For that, results of the largest RCTs (REMOTE-CAT, SERIC AIS, RESIST [14] and RICAMIS), which all include IS patients despite of the acute applied treatment, would be of enormous interest.

Only one RCT applied RIC manually [21], but one out of three patients fully complete the cycles. Using an automated RIC device allows that RIC can be continued once the patient arrives to the stroke care unit and the full dose can be administered. For that, most of the RTCs are currently using automatic devices to apply RIPerC. Concretely, 14 out of 17 new trials use automatic devices. Another important issue is the number of cycles and the place of application. Most RIC trials in Cardiology [25, 26] and the first trials in IS used the four-cycle protocol, probably due to literature tradition and preclinical studies. Preconditioning was first demonstrated in a dog model of myocardial ischemia using a four-cycle protocol [27]. Afterwards, both RIC before ischemia [28] and RIC during ischemia were first documented using the same protocol [29]. The neutral clinical results of Hougaard et al. [21] and Pico et al. [20] trials arise the need to increase the RIC stimulus and repetitions. Recent studies in preclinical models also addressed it to optimize the efficacy and optimal duration of RIPerC [30]. In a rat model of cerebral ischemia, repeated remote post-conditioning during 14 days after reperfusion significantly decrease the volume of infarction [31]. There are some promising experiences in chronic postconditioning among intracranial stenosis patients [32] and patients with cerebral small-vessel disease [33] using five-cycle protocol. Currently, on-going REMOTE-CAT and RESIST trials use a five-cycle RIC protocol. Moreover, combination of RIPerC and postconditioning during 4 to 7 days is assessed in the RESIST trial [14], ReCAST-2 [17], REPOST [16] and SERIC-AIS trial. Although, the volume of muscle mass affects the efficacy of the RIC intervention [34], only one study proposed RIC application in a leg [20]. It has been described that one in four IS patients has silent peripheral arterial disease [35], for that it has suggested that the upper arm would be the best location because of safety reasons. One and two-limb conditioning were equally protective according to preclinical models [30]. At present, how the neuroprotective stimulus is transferred or its mechanisms of actions in the brain are not fully understood [36], but it is known that the translation of the RIC sensory signal to the brain is crucial [37] and RIC should be applied in the non-affected arm.

A prehospital administration of RIC in the ambulance transportation was first proposed by Hougard et al. [21] and it is established in REMOTE-CAT and RESIST [14] trials. RIC effects are time-dependent, so early initiation of RIC is fundamental [38].

Pre-hospital screening scales should be used during transportation and RIPerC application to correctly randomize and recruit IS patients. When the Face Arm Speech Test was used, an increased proportion of patients with transient symptoms in the intervention group was observed [21]. It was not clear whether it was a RIC’s effect or there was a bias in the selection. At present, both REMOTE-CAT and RESIST [14] trials have a pre-hospital screening performed by RACE and PreSS scores, respectively. Patients should be properly balanced using prehospital stroke scores.

Recently, it has been reported that RIC improves the clinical evolution of myocardial infarction and it reduces the final lesion size [25, 39]; but a recent large RCT, with more than 5000 patients, reported no effects on clinical outcomes [40]. Cerebral and heart ischemia might differ on its own characteristics [41], because IS has a variety of pathogenic mechanisms not present in heart ischemia. The rupture or erosion of vulnerable plaques in coronary arteries are the most common cause of heart ischemia [42], while the embolism from arterial or heart sources is the main cause of IS [43]. Altogether, we would anticipate that underlying RIC mechanisms and clinical outcomes in IS patients will be different and the expected results of the currents RCTs are promising.

### Implications for future research

Currently, there are some on-going randomized clinical trials that will provide valuable information on RIPerC in ischemic stroke patients. However, future studies should carefully examine patient recruitment, RIPerC application settings, proper outcome measurements and neuroimaging follow-up protocols. All optimization and efforts will improve the current knowledge and address new medical strategies and management of stroke patients.

According to the RESCUE BRAIN study [20], the application of RIC during/after partial or complete reperfusion was futile, and it did not reduce the consequences of reperfusion injury. So, this might suggest that RIC should be applied differently. In this line, preclinical data and results from pilot studies showed that RIC should be applied as soon as possible, preferable during patient transportation (prehospital setting, ambulance) to a Hospital, in order to avoid the penumbral tissue recruitment and extend the time window for further application of reperfusion therapies. In this context, an early triage and stratification of the patients using prehospital scales are essential, and it will also help in the randomization process of the clinical trials (REMOTE-CAT, NCT03375762; RESIST [14]). The accuracy of prehospital scales is fundamental to identify or confirm a possible early prehospital treatment effect, like it was suspected in previous studies [21]. For that, the initial use of prehospital scales is a strong recommendation along RIPerC application in a prehospital setting and/or as soon as stroke symptoms are detected.

Automatic devices should be used to ensure completion of cycles and to document the treatment compliance. Another reason for the futile results of RESCUE BRAIN [20] study and the study of Hougaard et al. [21] would be that the 4 × 5 cycles of RIPerC stimulus was not sufficient. To overcome this issue, increasing up to five cycles and/or the stimulus repetition twice daily for the first 5 to 7 days would be an improvement. In the other hand, better selection of included patients in clinical trials can boost patient stratification.

Collateral status correlates with stroke severity and reperfusion outcomes, due to their ability to restrict the growth of penumbral territory [44]. Although the underlying mechanisms of RIC are still not fully known, some recent preclinical studies have showed an enhancement of collateral circulation [45, 46]. For that, the role of collaterals is essential in large vessel occlusion (LVO) patients, whom are also candidates to undergo mechanical thrombectomy [47, 48] in admitted hospital, or they are candidates to be transferred to a Comprehensive Stroke center. Altogether, LVO patients would be a group of special interest to study the RIC effects.

Recent published data have highlighted that RIC is safe and feasible [17–21, 49] similarly to RCTs involving patients with myocardial infarction [25]. For that, the main outcomes of the ongoing and future RCTs on RIPerC have a strong clinical interest. According to stroke treatment academic industry roundtable (STAIR) recommendations [50], 24-h NIHSS, 7-days mRS and 90-days mRS should be considered to be the standard clinical endpoints in acute stroke trials. Follow-up infarct volume on brain imaging is also informative, based on preclinical data that reported an effect of RIC on final brain infarction volume when it was used alone or in combination with alteplase [24]. This is recommended by both STAIR [50] and The Stroke Imaging Research (STIR) group [47]. More concretely, STIR estimated that sample sizes based on lesion volumes should be about one fourth of those based on mRS [51], so the imaging endpoint has the advantage of requiring smaller sample size. Finally, the understanding of RIPerC mechanisms and its neuroprotective role will be a key and animal models studies surely encourage better refined and translation into humans for the treatment of ischemic stroke.

## Conclusions

The summary of the completed and ongoing RCTs on RIPerC in IS patients shows that RIC can be initiated during pre-hospital transport, and it can be used alone or in combination with current recanalization therapies. RIPerC has the advantages of simplicity, safety, feasibility and affordability. The exact time window and the most effective neuroprotective RIC protocol are still not fully determined. Stroke preclinical animal models and RIC research are needed, both will also contribute to define the RIC molecular effects. Finally, ongoing RCTs will provide new information on the effect of RIPerC in IS patients, the optimal RIC protocol application and the underlying RIPerC mechanisms.

## Data Availability

The data that support the findings of this study are available from the corresponding author upon reasonable request.

## Abbreviations

IS: Ischemic stroke
IT: Ischemic tolerance
mRIC: Manual remote ischemic conditioning
RCT: Randomized clinical trials
RIC: Remote ischemic conditioning
RIPerC: Remote ischemic preconditioning
Rt-PA: Recombinant tissue plasminogen activator
VEGF: Vascular endothelial growth factor

## References

[1] Mozaffarian D, Benjamin EJ, Go AS, Arnett DK, Blaha MJ, Cushman M (2016). Executive summary: heart disease and stroke statistics--2016 update: a report from the American Heart Association. Circulation.

[2] Pandian JD, Gall SL, Kate MP, Silva GS, Akinyemi RO, Ovbiagele BI (2018). Prevention of stroke: a global perspective. Lancet.

[3] Purroy F, Vena A, Forne C, de Arce AM, Davalos A, Fuentes B (2019). Age- and sex-specific risk profiles and in-hospital mortality in 13,932 Spanish stroke patients. Cerebrovasc Dis.

[4] Lees KR, Emberson J, Blackwell L, Bluhmki E, Davis SM, Donnan GA (2016). Effects of Alteplase for acute stroke on the distribution of functional outcomes: a pooled analysis of 9 trials. Stroke.

[5] Emberson J, Lees KR, Lyden P, Blackwell L, Albers G, Bluhmki E (2014). Effect of treatment delay, age, and stroke severity on the effects of intravenous thrombolysis with alteplase for acute ischaemic stroke: a meta-analysis of individual patient data from randomised trials. Lancet.

[6] Goyal M, Menon BK, van Zwam WH, Dippel DW, Mitchell PJ, Demchuk AM (2016). Endovascular thrombectomy after large-vessel ischaemic stroke: a meta-analysis of individual patient data from five randomised trials. Lancet.

[7] Chamorro A, Dirnagl U, Urra X, Planas AM (2016). Neuroprotection in acute stroke: targeting excitotoxicity, oxidative and nitrosative stress, and inflammation. Lancet Neurol.

[8] O'Collins VE, Macleod MR, Donnan GA, Horky LL, van der Worp BH, Howells DW (2006). 1,026 experimental treatments in acute stroke. Ann Neurol.

[9] Percie du Sert N, Alfieri A, Allan SM, Carswell HV, Deuchar GA, Farr TD (2017). The IMPROVE guidelines (Ischaemia models: procedural refinements of in vivo experiments). J Cereb Blood Flow Metab.

[10] Hausenloy DJ, Yellon DM (2011). The therapeutic potential of ischemic conditioning: an update. Nat Rev Cardiol.

[11] Hess DC, Blauenfeldt RA, Andersen G, Hougaard KD, Hoda MN, Ding Y (2015). Remote ischaemic conditioning-a new paradigm of self-protection in the brain. Nat Rev Neurol.

[12] Gidday JM (2006). Cerebral preconditioning and ischaemic tolerance. Nat Rev Neurosci.

[13] Moher D, Liberati A, Tetzlaff J, Altman DG, Group P (2009). Preferred reporting items for systematic reviews and meta-analyses: the PRISMA statement. J Clin Epidemiol.

[14] Blauenfeldt RA, Hjort N, Gude MF, Behrndtz AB, Fisher M, Valentin JB (2020). A multicentre, randomised, sham-controlled trial on REmote iSchemic conditioning in patients with acute STroke (RESIST) - rationale and study design. Eur Stroke J.

[15] Pico F, Rosso C, Meseguer E, Chadenat ML, Cattenoy A, Aegerter P (2016). A multicenter, randomized trial on neuroprotection with remote ischemic per-conditioning during acute ischemic stroke: the REmote iSchemic Conditioning in acUtE BRAin INfarction study protocol. Int J Stroke.

[16] Landman T, Schoon Y, Warle M, De Leeuw FE, Thijssen D (2019). The effect of repeated remote ischemic postconditioning on infarct size in patients with an ischemic stroke (REPOST): study protocol for a randomized clinical trial. Trials.

[17] England TJ, Hedstrom A, O'Sullivan S, Donnelly R, Barrett DA, Sarmad S (2017). RECAST (remote ischemic conditioning after stroke trial): a pilot randomized placebo controlled phase II trial in acute ischemic stroke. Stroke.

[18] Zhao W, Che R, Li S, Ren C, Li C, Wu C (2018). Remote ischemic conditioning for acute stroke patients treated with thrombectomy. Ann Clin Transl Neurol.

[19] Che R, Zhao W, Ma Q, Jiang F, Wu L, Yu Z (2019). rt-PA with remote ischemic postconditioning for acute ischemic stroke. Ann Clin Transl Neurol.

[20] Pico F, Lapergue B, Ferrigno M, Rosso C, Meseguer E, Chadenat ML (2020). Effect of in-hospital remote ischemic perconditioning on brain infarction growth and clinical outcomes in patients with acute ischemic stroke: the RESCUE BRAIN randomized clinical trial. JAMA Neurol.

[21] Hougaard KD, Hjort N, Zeidler D, Sørensen L, Nørgaard A, Hansen TM (2014). Remote ischemic perconditioning as an adjunct therapy to thrombolysis in patients with acute ischemic stroke: a randomized trial. Stroke.

[22] Pico F, Laperque M, Ferrigno M, Chadenat ML, Bourdain F, Meseguer E (2019). The RESCUE BRAIN trial: a french multicenter randomized trial on neuroprotection with lower limb ischemic per-conditioning in the acute phase of cerebral infarction. Eur Stroke J.

[23] Perez de la Ossa N, Carrera D, Gorchs M, Querol M, Millan M, Gomis M (2014). Design and validation of a prehospital stroke scale to predict large arterial occlusion: the rapid arterial occlusion evaluation scale. Stroke.

[24] Hoda MN, Siddiqui S, Herberg S, Periyasamy-Thandavan S, Bhatia K, Hafez SS (2012). Remote ischemic perconditioning is effective alone and in combination with intravenous tissue-type plasminogen activator in murine model of embolic stroke. Stroke.

[25] Man C, Gong D, Zhou Y, Fan Y (2017). Meta-analysis of remote ischemic conditioning in patients with acute myocardial infarction. Sci Rep.

[26] Pryds K, Nielsen RR, Jorsal A, Hansen MS, Ringgaard S, Refsgaard J (2017). Effect of long-term remote ischemic conditioning in patients with chronic ischemic heart failure. Basic Res Cardiol.

[27] Murry CE, Jennings RB, Reimer KA (1986). Preconditioning with ischemia: a delay of lethal cell injury in ischemic myocardium. Circulation.

[28] Birnbaum Y, Hale SL, Kloner RA (1997). Ischemic preconditioning at a distance: reduction of myocardial infarct size by partial reduction of blood supply combined with rapid stimulation of the gastrocnemius muscle in the rabbit. Circulation.

[29] Schmidt MR, Smerup M, Konstantinov IE, Shimizu M, Li J, Cheung M (2007). Intermittent peripheral tissue ischemia during coronary ischemia reduces myocardial infarction through a KATP-dependent mechanism: first demonstration of remote ischemic perconditioning. Am J Physiol Heart Circ Physiol.

[30] Johnsen J, Pryds K, Salman R, Lofgren B, Kristiansen SB, Botker HE (2016). The remote ischemic preconditioning algorithm: effect of number of cycles, cycle duration and effector organ mass on efficacy of protection. Basic Res Cardiol.

[31] Ren C, Wang P, Wang B, Li N, Li W, Zhang C (2015). Limb remote ischemic per-conditioning in combination with post-conditioning reduces brain damage and promotes neuroglobin expression in the rat brain after ischemic stroke. Restor Neurol Neurosci.

[32] Meng R, Asmaro K, Meng L, Liu Y, Ma C, Xi C (2012). Upper limb ischemic preconditioning prevents recurrent stroke in intracranial arterial stenosis. Neurology.

[33] Wang Y, Meng R, Song H, Liu G, Hua Y, Cui D (2017). Remote ischemic conditioning may improve outcomes of patients with cerebral small-vessel disease. Stroke.

[34] Hess DC, Hoda MN, Bhatia K (2013). Remote limb perconditioning [corrected] and postconditioning: will it translate into a promising treatment for acute stroke?. Stroke.

[35] Purroy F, Coll B, Oro M, Seto E, Pinol-Ripoll G, Plana A (2010). Predictive value of ankle brachial index in patients with acute ischaemic stroke. Eur J Neurol.

[36] Heusch G, Botker HE, Przyklenk K, Redington A, Yellon D (2015). Remote ischemic conditioning. J Am Coll Cardiol.

[37] Donato F, Rompani SB, Caroni P (2013). Parvalbumin-expressing basket-cell network plasticity induced by experience regulates adult learning. Nature.

[38] Koch S, Gonzalez N (2013). Preconditioning the human brain: proving the principle in subarachnoid hemorrhage. Stroke.

[39] Botker HE, Kharbanda R, Schmidt MR, Bottcher M, Kaltoft AK, Terkelsen CJ (2010). Remote ischaemic conditioning before hospital admission, as a complement to angioplasty, and effect on myocardial salvage in patients with acute myocardial infarction: a randomised trial. Lancet.

[40] Hausenloy DJ, Kharbanda RK, Moller UK, Ramlall M, Aaroe J, Butler R (2019). Effect of remote ischaemic conditioning on clinical outcomes in patients with acute myocardial infarction (CONDI-2/ERIC-PPCI): a single-blind randomised controlled trial. Lancet.

[41] Soler EP, Ruiz VC (2010). Epidemiology and risk factors of cerebral ischemia and ischemic heart diseases: similarities and differences. Curr Cardiol Rev.

[42] White HD, Chew DP (2008). Acute myocardial infarction. Lancet.

[43] Campbell BCV, De Silva DA, Macleod MR, Coutts SB, Schwamm LH, Davis SM (2019). Ischaemic stroke. Nat Rev Dis Primers.

[44] Kimmel ER, Al Kasab S, Harvey JB, Bathla G, Ortega-Gutierrez S, Toth G (2019). Absence of collaterals is associated with larger infarct volume and worse outcome in patients with large vessel occlusion and mild symptoms. J Stroke Cerebrovasc Dis.

[45] Zhang Y, Ma L, Ren C, Liu K, Tian X, Wu D (2019). Immediate remote ischemic postconditioning reduces cerebral damage in ischemic stroke mice by enhancing leptomeningeal collateral circulation. J Cell Physiol.

[46] Kitagawa K, Saitoh M, Ishizuka K, Shimizu S (2018). Remote limb ischemic conditioning during cerebral ischemia reduces infarct size through enhanced collateral circulation in murine focal cerebral ischemia. J Stroke Cerebrovasc Dis.

[47] Warach SJ, Luby M, Albers GW, Bammer R, Bivard A, Campbell BC (2016). Acute stroke imaging research roadmap III imaging selection and outcomes in acute stroke reperfusion clinical trials: consensus recommendations and further research priorities. Stroke.

[48] Berkhemer OA, Jansen IG, Beumer D, Fransen PS, van den Berg LA, Yoo AJ (2016). Collateral status on baseline computed tomographic angiography and intra-arterial treatment effect in patients with proximal anterior circulation stroke. Stroke.

[49] Kate M, Brar S, George U, Rathore S, Butcher K, Pandian J (2019). Self- or caregiver-delivered manual remote ischemic conditioning therapy in acute ischemic stroke is feasible: the Early Remote Ischemic Conditioning in Stroke (ERICS) trial. Wellcome Open Res.

[50] Jovin TG, Albers GW, Liebeskind DS, Consortium SI (2016). Stroke treatment academic industry roundtable: the next generation of endovascular trials. Stroke.

[51] Whitehead J, Bolland K, Valdes-Marquez E, Lihic A, Ali M, Lees K (2009). Using historical lesion volume data in the design of a new phase II clinical trial in acute stroke. Stroke.

